# Association of homocysteine and methylene tetrahydrofolate reductase (MTHFR C677T) gene polymorphism with coronary artery disease (CAD) in the population of North India

**DOI:** 10.1590/S1415-47572010005000026

**Published:** 2010-06-01

**Authors:** Rajneesh Tripathi, Satyendra Tewari, Prabhat Kumar Singh, Sarita Agarwal

**Affiliations:** 1 Department of Genetics, Sanjay Gandhi Post Graduate Institute of Medical Sciences, LucknowIndia; 2 Department of Cardiology, Sanjay Gandhi Post Graduate Institute of Medical Sciences, LucknowIndia; 3 Department of Anaesthesiology, Sanjay Gandhi Post Graduate Institute of Medical Sciences, LucknowIndia

**Keywords:** angiography, CAD, homocysteine, MTHFR polymorphism

## Abstract

The implications of the methylene tetrahydrofolate reductase (MTHFR) gene and the level of homocysteine in the pathogenesis of coronary artery disease (CAD) have been extensively studied in various ethnic groups. Our aim was to discover the association of MTHFR (C677T) polymorphism and homocysteine level with CAD in north Indian subjects. The study group consisted of 329 angiographically proven CAD patients, and 331 age and sex matched healthy individuals as controls. MTHFR (C677T) gene polymorphism was detected based on the polymerase chain reaction and restriction digestion with *Hinf*I. Total homocysteine plasma concentration was measured using immunoassay. T allele frequency was found to be significantly higher in patients than in the control group. We found significantly elevated levels of mean homocysteine in the patient group when compared to the control group (p = 0.00). Traditional risk factors such as diabetes, hypertension, smoking habits, a positive family history and lipid profiles (triglyceride, total cholesterol, HDL-cholesterol, LDL-cholesterol, VLDL-cholesterol), were found significantly associated through univariate analysis. Furthermore, multivariable logistics regression analysis revealed that CAD is significantly and variably associated with diabetes, hypertension, smoking, triglycerides and HDL-cholesterol. Our findings showed that MTHFR C677T polymorphism and homocysteine levels were associated with coronary artery disease in the selected population.

## Introduction

Coronary artery disease (CAD) is the consequence of atherosclerotic plaque disposition on the coronary artery wall. Its manifestation depends on interactions between environmental and genetic risk factors (Andreassi *et al.*, 2003). Individual susceptibility to the disease has been associated with functional allelic variation. Thus, the identification of gene polymorphisms relating to the formation of atherosclerotic plaques, and consequently, thrombi, may contribute towards developing early diagnostic methods and guiding preventive procedures (Arruda *et al.*, 1998; Doevendans *et al.*, 2001; Andreassi *et al.*, 2003). The gene that encodes methylene tetrahydrofolatereductase enzyme (MTHFR), which is involved in the metabolism of homocysteine (Hcy), is of great interest in clinical practice (Robinson *et al.*, 1995; Hofmann *et al.*, 2001; Andreassi *et al.*, 2003). Hcy, an amino acid derived from protein catabolism, is present in the plasma in several forms, with the greatest proportion (70%) bound to albumin (Doevendans *et al.*, 2001). High levels of Hcy (hyperhomocysteinemia) have been identified as a risk factor for atherosclerosis (Bova *et al.*, 1999; Hankey and Eikelboom, 1999; Lobo *et al.*, 1999; Doevendans *et al.*, 2001; Andreassi *et al.*, 2003). The mechanism for the vascular lesions induced by hyperhomocysteinemia remains unclear. Experimental evidence suggests that Hcy facilitates the vascular oxidative process, thereby altering the coagulation system, and reduces the vasomotor regulation of the endothelium (Welch *et al.*, 1997; Tyagi, 1998; Hankey and Eikelboom, 1999; Hofmann *et al.*, 2001). The aim of this study was to assess the relationship between MTHFR gene polymorphism and plasma homocysteine level with the traditional risk factors of coronary artery disease in a north Indian population.

## Material and Methods

###  Study subjects

Patients, with evidence of more than 50% stenosis in coronary arteries, a past history of prior angioplasty, or CAD by-pass grafting, were included in the present study. The patients were recruited from the outpatient and inpatient services of the Department of Cardiology, SGPGIMS during the period February, 2006 to November, 2008. The study group included angiographically proven CAD patients. The control group consisted of individuals without a CAD background and with negative results in treadmill-stress tests. The two groups were age and sex matched. An individual clinical background entailing the presence of diabetes, hypertension, smoking habits, CAD family history, body-mass index and complete lipid profile (serum triglycerides, total cholesterol, HDL, LDL and VLDL) were furnished by members of both the patient and control groups independently. Diabetes and hypertension was diagnosed in patients and controls, based on prior medical records or standard clinical examination and tests, according to the standard definition described by Fauci *et al.* (2008). Habitual smoking was defined as prevailing at least one year before CAD onset. A family history of CAD was defined based on its presence in first-degree relatives. Body-mass index was calculated by dividing the weight in kilograms by the square of the height in meters (Fouci *et al.*, 2008). Patients and controls included in the study were natives of Uttar Pradesh. Exclusion criteria were cardiomyopathy, a febrile condition, rheumatic heart disease, congenital heart disease and systemic disorders. A prior written informed consent was obtained from patients and controls. The study was approved by an institutional ethics committee.

###  DNA preparation and genotyping

**Figure 1:**
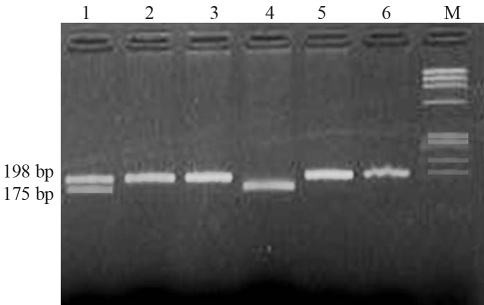
Detection of MTHFR polymorphism. Lanes 2, 3, 5 and 6 showing wild type (CC) genotype, lane 1 showing mutant heterozygous (CT) genotype and lane 4 showing mutant homozygous (TT) genotype, M showing molecular weight Marker.

Genomic DNA was extracted from EDTA peripheral blood leukocytes by the standard phenol-chloroform method (Poncz *et al.*, 1982). The quality of DNA was checked through 0.8% agarose (Sigma, USA) gel electrophoresis, and quantification was done on a UV spectrophotometer (Specgene Ltd, UK). Synthetic oligonucleotides were acquired from Genetix, France. Primers (Forward 5'TGA AGG AGA AGG TGT CTG CGG GA3', Reverse 5'AGG ACG GTG CGG TGA GAG TG3') for exon 4 of the MTHFR gene were used as described by Frost *et al.* (1995). Amplification was done in an automated thermocycler (BioRad PTC100, USA). The mixture was initially denatured at 94 °C for 4 min, followed by 30 cycles with 30 s at 94 °C, 45 s at 62 °C, 45 s at 72 °C and a 12 min final extension at 72 °C. PCR products were checked on 2% of agarose gels followed by staining with 1 μg/mL of ethidium bromide. . The amplified PCR products of 198 bp were digested with *Hinf*I (NEB UK) restriction enzyme according to manufacturer's specifications. The restriction digestion products were separated on a 3% agarose gel and visualized by ethidium bromide staining (Figure 1).

###  Biochemical analyses

Fasting venous blood from patients and controls were collected in plain vials in order to analyze triglyceride (TG) levels. These levels were measured by lipoprotein lipase-peroxidase (Fossati and Prencipe*,* 1982), those of total cholesterol (TC) by cholesterol oxidase (Allain *et al.*, 1974) and those of high density lipoprotein (HDL) by phosphotungstate magnesium chloride methodology (Lopes-Virella *et al.*, 1977). Very low density lipoprotein (VLDL) and low density lipoprotein (LDL) levels were calculated indirectly by means of the Friedwald formula (Friedewald *et al.*, 1972) as (VLDL-C = TG/5 provided TG = 400 mg/dL) and LDL-C = TC – (HDL-C + VLDL-C). Total plasma homocysteine levels were determined in 87 patients and 79 controls by enzyme linked immunoassay (Microplate EIA Homocysteine BioRad, USA). The assay method was based on enzymatic conversion of homocysteine to S-adenosyl homocysteine, followed by quantification of S-adenosyl-L-homocysteine by an enzyme- linked immunoassay (Frantzen *et al.*, 1998).

###  Statistical analysis

Genotype and allele frequencies in CAD and control groups were compared by Chi square testing. The characteristics of patients and controls were evaluated by comparing biochemical findings using the Student t-test. Additionally, we performed multiple logistic regression model testing on the interaction of genotype and different classical CAD risk factors. All analyses were performed using SPSS v.11.5 (SPSS Inc., Chicago, USA) statistical analysis software. A two-tailed p value of p < 0.05 was considered statistically significant.

## Results

The study group included 329 angiographically proven CAD patients [80 with myocardial infarction (MI), and the remaining 249 with no past reference], and 331 individuals as controls with no clinical history of CAD. The clinical and demographic data of patients and controls are presented in Table 1. Classical risk factors, viz., diabetes, hypertension, smoking and family history were significantly higher in the CAD group than in the control. The value of HDL- cholesterol was significantly lower in CAD patients than in controls, although serum triglyceride, total cholesterol, LDL-cholesterol, VLDL-cholesterol and homocysteine levels were found to be significantly higher in the former.

Allele and genotype frequencies in both patient and control group are described in Table 2. The distribution of genotypes within each group was in Hardy-Weinberg equilibrium. We found the prevalence of the CT genotype to be 11.5% in the control and 18.2% in the patient group, whereas almost similar mutant homozygous frequencies (1.5% and 2.8%) were observed in controls and patients, respectively. T allele frequency was significantly higher in the patient group (11.9%) than the control (7.3%) (p = 0.004, OR 1.75 95% CI 1.16-2.55). On compiling a subgroup of MI and non-MI patients, we found that 63 out of 80 MI patients (78.6%) presented a CC genotype, 13 (16.25%) a CT genotype, and 4 (5.0%) were homozygous for the T allele (TT genotype). C and T allele frequencies were 87% [139] and 13% [21], respectively, in the MI patient group. This indicates a significant association of the T allele with myocardial infarction in patients in contrast to control [p = 0.016 (Fisher exact), OR 1.93 (95% CI 1.08-3.44)].

When we assessed the severity of the disease in terms of the number of vessels involved in CAD and MTHFR polymorphism, interestingly we found no variation in T allele prevalence in either one, two or three vessels [12.7%, 10.0% and 11.0%, respectively] with stenosis in a CAD patient. Similarly findings were observed with homocysteine levels (Table 3). Significantly high levels of homocysteine were observed in the patient group as compared to the control, irrespective of the genotypes involved (CC, CT and TT) (Table 3).

On performing multiple logistic regression analysis; to see the influence of various risk factors on CAD precipitation (Table 4), we found that, in the case of diabetes, hypertension, smoking, triglyceride and HDL-cholesterol, this was significantly variable for CAD. We additionally observed that an increase to 10 mg/dL in Triglyceride, Total cholesterol and LDL-cholesterol, will increase the risk of CAD by 1.3 times, 1.01 times and 1.08 times, respectively, whereas a 10 mg/dL decrease in HDL-cholesterol leads to a 2.5 times greater risk of CAD. This indicates that HDL-C is more crucial for CAD. Furthermore a concurrent increase in MTHFR CT and TT genotypes leads to an even greater CAD risk (1.3 times and 1.2 times, respectively) than CC genotype.

## Discussion

The implication of the MTHFR gene in CAD pathogenesis has been extensively studied in several ethnic groups. We found a significantly higher frequency of T alleles in CAD patients than in controls. OR in this group was 1.75. Other epidemiological studies that estimated the risk of CAD associated with the T allele showed conflicting results. Notwithstanding, Kerkeni *et al.* (2006) and Alam *et al.* (2008) found this substitution to be a significant risk factor. On summarizing the results from 8 studies, Kluijtmans *et al.* (1996) encountered no significant difference for the T allele, although they reported higher frequencies (31.8% and 29% in patient and control group respectively).

We found the T allele to be significantly associated with myocardial infarction (p = 0.016, OR1.93, 95% CI 1.08-3.44). This finding is consistent with a previous report (Gülec *et al.*, 2001) in which MTHFR C677T transition was found to be a risk factor for premature MI. On the other hand, other authors ( Anderson *et al.*, 1997; Hsu *et al.*, 2001) reported insignificant differences for the T allele between MI subjects and the control group. We observed almost equal frequencies for the CC, CT and TT genotypes, irrespective of one-, two- or three-vessel-stenosis, Our findings are in accordance with previous reports (Van Bockxmeer *et al.*, 1997; Kerkeni *et al.*, 2006; Rassoul *et al.*, 2008), in which insignificant association was observed. Nevertheless, for Morita *et al.* (1997) the association was significant. Furthermore, we noted that the severity of the disease is independent of homocysteine levels. Likewise, Wang *et al.* (1999) found no correlation between the level of homocysteine and severity of the disease, whereas Rassoul *et al.* (2008), on the contrary, discovered a positive association.

In our study, the average homocysteine level was significantly higher in the patient group than in the control. This is in agreement with observations by other investigators (Evans *et al.*, 1997; Kerkeni *et al.*, 2006). The finding of a significant correlation between 5,10 methylene tetrahydrofolate reductase enzyme activity and MTHFR genotypes (Frosst *et al.*, 1995) (70% reduced activity in the TT genotype and 35% reduced activity in the CT genotype) implies elevated homocysteine levels in these two genotypes.

A previous study by Alam *et al.* (2008) on 100 coronary artery cases and 100 controls, also from North India, demonstrated the positive association of MTHFR (C677T) gene polymorphism. The present study is in agreement, although with a larger-sized sample, and undertaken with certain restrictions, such as using a single MTHFR genetic marker (C677T). Thus, further polymorphic MTHFR markers need to be evaluated, together with the other genetic markers involved in the Homocysteine pathway for predisposition to the disease.

Coronary artery disease is a complex disorder where environmental and genetic markers both play an important role.

## Figures and Tables

**Table 1:** Characteristics of controls and patients.

Characteristics	Controls	CAD Patients	p-value
N	331	329	-
Age	53.18 ± 9.18*	57.09 ± 9.66*	0.217
Sex (M:F)	245:86	260:69	0.142
BMI	25.12 ± 3.12*	25.54 ± 2.97*	0.077
Diabetic: nondiabetic	82:249	142:187	0.000
Hypertensive: nonhypertensive	76:255	156:173	0.000
Smoker: nonsmoker	33:299	66:263	0.000
Positive family history: negative family history	04:329	13:316	0.028**
Triglyceride (45-150 mg/dL)	95.75 ± 27.95*	165.04 ± 81.33*	0.000
Total Cholesterol (125-250 mg/dL)	154.70 ± 39.73*	170.29 ± 48.56*	0.000
HDL-cholesterol (23-60 mg/dL)	38.34 ± 7.79*	36.51 ± 7.01*	0.002
LDL-cholesterol (92-148 mg/dL)	97.82 ± 31.72*	103.99 ± 43.67*	0.039
VLDL-cholesterol (10-30 mg/dL)	19.59 ± 6.65*	29.80 ± 13.90*	0.000
Homocysteine (5-15 μM/L)	11.11 ± 4.39	16.05 ± 8.51	0.000

*Mean ± SD, ** p-value (Yates correction).

**Table 2:** Distribution of genotype and allele frequencies in patients and controls.

Genotype/Allele	Control (N = 331)	Patient (N = 329)	OR	p-value	95% CI
CC	288 (87.0%)	260 (79.0%)			
CT	38 (11.5%)	60 (18.20%)		0.054	
TT	5 (1.5%)	9 (2.8%)			
C allele	614 (92.7%)	580 (88.1%)	1.75	0.004	1.16-2.55
T allele	48 (7.3%)	72 (11.9%)			

**Table 3:** Plasma homocysteine levels in patients and controls.

	Homocysteine level* controls (N = 79)	Homocysteine level* patients (N = 87)	p-value
CC Genotype	11.86 ± 3.88 (66)	14.42 ± 8.62 (56)	0.000
CT Genotype	11.094 ± 7.11 (9)	18.64 ± 7.06 (25)	0.000
TT Genotype	16.1788 ± 3.96 (4)	20.59 ± 10.07 (6)	0.000
1 vessel	-	17.60 ± 9.47 (32)	
2 vessel	-	15.15 ± 6.56 (21)	0.437
3 vessel	-	15.15 ± 8.63 (34)	

*Values in (Mean ± SD); () number.

**Table 4:** Multivariable logistic regression analysis between controls and patients.

Variable	B	p-value	OR	95% CI
BMI	0.049	0.168	1.050	0.980-1.125
Diabetes	0.844	0.000	2.326	1.480-3.655
Hypertension	0.923	0.000	2.490	1.589-3.901
Smoking	0.713	0.017	2.040	1.136-3.664
Family history	1.004	0.172	2.728	0.649-11.45
Triglyceride	0.033	0.000	1.034	1.021-1.05
Total cholesterol	0.002	0.796	1.002	0.984-1.021
HDL-cholesterol	-0.110	0.000	0.895	0.861-0.931
LDL-cholesterol	0.011	0.260	1.011	0.992-1.031
VLDL-cholesterol	-0.022	0.450	0.979	0.926-1.035
MTHFR CT genotype	0.295	0.319	1.343	0.752-2.396
MTHFR TT genotype	0.236	0.751	1.226	0. 295-5.423
